# Supplementary material for: A death pheromone, oleic acid, triggers hygienic behavior in honey bees (Apis mellifera L.)
Source: Sci Rep. 2018 Apr 9;8:5719. doi: 10.1038/s41598-018-24054-2 (PMC5890279; doi:10.1038/s41598-018-24054-2)
Supplement: Supplementary file 1 — Supplementary tables S1–5 [file 41598_2018_24054_MOESM1_ESM.pdf]

A death pheromone, oleic acid, triggers hygienic behavior in honey bees (*Apis mellifera* L.)

Alison McAfee, Abigail Chapman, Immacolata Iovinella, Ylonna Gallagher-Kurtzke, Troy F. Collins, Heather Higo, Lufiani L. Madilao, Paolo Pelosi, and Leonard J. Foster

Table of Contents

Table S1	Page 2
Table S2	Page 2
Table S3	Page 3
Table S4	Page 3
Table S5	Page 3

Table S1. Log10 transformed SPME GC-MS peak areas for N = 5 brood from each stage from 5 different colonies (only peaks with > 4,000 cts apex intensity are displayed)

			Larvae		Prepupae		Pupae	
**Peak #**	**Retention time (min)**	**Proposed identity**	**Ave**	**St. Dev.**	**Ave**	**St. Dev.**	**Ave**	**St. Dev.**
1	2.49	Thiobis-methane	6.50	0.13	6.48	0.14	6.53	0.12
2	2.97	2-propanone	4.89	0.16	5.49	0.10	5.81	0.12
3	3.04	Octane	4.99	0.33	4.07	0.27	4.08	0.53
4	3.75	Isopropanol	5.81	0.16	4.61	0.23	5.11	0.25
5	10.23	Isoamyl acetoacetate	4.98	0.50	3.93	0.50	3.82	0.36
6*	11.78	β-cis-Ocimene	6.22	0.39	6.43	0.23	5.73	0.15
7	13.81	β-octahydroindoloquinolizine	4.88	0.10	4.69	0.22	4.77	0.21
8	17.17	Acetic acid	4.66	0.67	3.58	0.19	3.96	0.16
9	19.94	Ethyl 4-(chloromethylene)-2,2-diphenyl-3-oxazoline-5-carboxylate	4.63	0.14	4.17	0.30	4.20	0.18
10	20.78	Propanoic acid	5.09	0.87	4.50	1.29	3.43	0.22
11	21.07	n/a	4.48	0.11	3.80	0.44	3.36	0.14
12	25.56	1,3-diphenyl-1-trimethylsilyloxy-1-pentene	4.20	0.36	3.75	0.29	3.68	0.18

*Compound identity confirmed as β-ocimene based on spectral matching and comparing the retention time to a standard.

Table S2. Log10 transformed hexane wash GC-MS peak areas for N = 5 brood from each stage from 5 different colonies (only peaks with > 4,000 cts apex intensity are displayed)

			Larvae		Prepupae		Pupae	
**Peak #**	**RT**	**Proposed identity**	**Ave.**	**St. Dev.**	**Ave.**	**St. Dev.**	**Ave.**	**St. Dev.**
1	14.78	Triacontane	6.24	0.21	6.02	0.10	6.46	0.07
2	14.93	2-methyl hexadecane	5.83	0.09	6.18	0.12	6.27	0.06
3	15.91	n-pentacosane	6.78	0.19	6.57	0.12	6.92	0.02
4	16.07	Heptadecane	6.41	0.11	6.90	0.10	6.84	0.04
5	17.27	Octacosane	7.04	0.09	7.29	0.09	7.64	0.02
6	17.47	1-eicosanol	6.82	0.11	7.47	0.09	7.56	0.03
7	18.09	Tricosane	5.44	0.10	6.08	0.09	6.58	0.02
8	18.31	Hexatriacontane	5.35	0.18	6.29	0.09	6.58	0.02
9	19.14	Nonacosane	6.50	0.06	6.83	0.12	7.42	0.03
10	19.41	2-methyl-octadecane	6.55	0.23	7.15	0.08	7.56	0.01
11	22.01	Tetracosane	6.07	0.07	6.13	0.10	6.67	0.07
12	22.41	2-methyl-eicosane	6.56	0.28	6.82	0.08	7.31	0.02
13	27.28	2-methyl-octadecane	6.24	0.27	6.42	0.14	6.84	0.05

Table S3. Comparing binding constants between our study and a previous report

		McAfee et al 2017		Guarna et al 2015	
**Protein**	**Ligand**	**IC_{50} (μM)**	**K_d**	**IC_{50} (μM)**	**K_d**
OBP16	Oleic acid	1.5	0.48	0.9	0.4
	Ocimene	2.3	0.74		
	Hexane	40	12.9		
	Phenylethyl acetate	40	12.9	8	3.7
	1-NPN	0.6	0.97		
OBP18	Oleic acid	0.3	0.13	0.1	0.04
	Ocimene	7	3.13		
	Hexane	>50			
	Phenylethyl acetate	>50	2.5	1.1	
	1-NPN	1.1	2.8		

Table S4. Odor toxicity assays

**Odor**	**Number with halted development**	**% with halted development**
Hexane	0	0
β-ocimene	0	0
Oleic acid	6	40
Mix	4	27

Table S5: Frequencies of brood ages across all front-way odor assays

	**Total brood**	**% Larvae**	**% Prepupae**	**% Pupae**
Hexane	822	10.5	60.1	29.4
Mix	853	12.5	62.1	25.3
Ocimene	817	6.2	63.8	30.0
Oleic acid	818	8.8	65.0	26.2
Phenethyl acetate	803	8.5	62.5	29.0
Overall	4113	9.3	62.7	28.0
